# Plant traits regulated metal(loid)s in dominant herbs in an antimony mining area of the karst zone, China

**DOI:** 10.1002/ece3.70212

**Published:** 2024-08-23

**Authors:** Zhongyu Du, Shufeng Wang, Wenli Xing, Liang Xue, Jiang Xiao, Guangcai Chen

**Affiliations:** ^1^ Research Institute of Subtropical Forestry Chinese Academy of Forestry Hangzhou China

**Keywords:** antimony mining, dominant species, ecological restoration, ecological stoichiometry, functional traits, metal(loid)s accumulation

## Abstract

Understanding how plant functional traits respond to mining activities and impact metal(loid)s accumulation in dominant species is crucial for exploring the driving mechanisms behind plant community succession and predicting the ecological restoration potential of these plants. In this study, we investigated four dominant herbaceous species (*Artemisia argyi*, *Miscanthus sinensis*, *Ficus tikoua*, and *Ageratina adenophora*) growing on antimony (Sb) mining sites (MS) with high Sb and arsenic (As) levels, as well as non‐mining sites (NMS). The aim was to analyze the variations in functional traits and their contribution to Sb and As concentrations in plants. Our results indicate that mining activities enhanced soil nitrogen (N) limitation and phosphorus (P) enrichment, while significantly reducing the plant height of three species, except for *F. tikoua*. The four species absorbed more calcium (Ca) to ensure higher tolerance to Sb and As levels, which is related to the activation of Ca signaling pathways and defense mechanisms. Furthermore, plant Sb and As concentrations were dependent on soil metal(loid) levels and plant element stoichiometry. Overall, these findings highlight the regulatory role of plant element traits in metal(loid) concentrations, warranting widespread attention and further study in the future.

## Graphical abstract

Four dominant species (*Artemisia argyi*, *Miscanthus sinensis*, *Ficus tikoua*, and *Ageratina adenophora*) were selected to investigate the functional traits variation and their contributions to Sb and As accumulation in high levels of antimony (Sb) and arsenic (As) sites (MS) and the non‐mining sites (NMS). Four species absorbed more Ca to ensure their higher tolerance to Sb and As, which is related to the Ca signal conduction startup of the defense mechanism. Plant Sb and As levels were not only dependent on plant height, but also plant N, P, and Ca concentration and stoichiometry. These findings emphasize the influence of mining activities on modifying plant adaptation strategies and highlight the regulation role of plant traits on metal(loid)s accumulation.

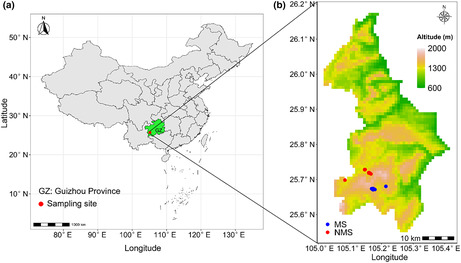

## 1 INTRODUCTION

Mining activities have significantly caused metal(loid)s pollution and extensive ecological damage (Guo et al., 2023; Li et al., 2014). Metal(loid)s in the soil can migrate through the soil‐plant‐human system, affecting agricultural product quality and posing risks to human health (Prasad et al., 2021). Ecological restoration of mining sites is crucial as it plays a vital role in preventing the further spread of metal(loid)s. The establishment and succession of plant communities are key approaches to restoring ecosystems damaged by mining activities, helping to improve damaged habitats and restore ecosystem functions (Ahirwal & Maiti, 2018). Restoring ecosystem function is a lengthy process influenced by vegetation adaptation to the environment, followed by plant growth and succession (Festin et al., 2019). Previous studies have given considerable attention to assessing biodiversity and ecosystem function in post‐mining sites (Bashirzadeh et al., 2022; Pitz et al., 2016). However, the understanding of how plants adapt to areas degraded by mining activities remains limited. Therefore, a deeper investigation into this aspect is necessary to develop effective restoration strategies.

Functional traits are essential for assessing plant adaption to environmental changes, encompassing morphological, chemical, physiological, and reproductive characteristics. Notably, leaf traits are typically linked to plant strategies associated with resource acquisition, utilization, and preservation (Song et al., 2019). These trait values exhibit variability not only within species but also among species, as well as across environmental gradients (Dong et al., 2020; Dwyer et al., 2014). Extensive research has explored the nature of leaf trait variation, demonstrating systematic relationships between leaf traits and climate (Cornwell et al., 2018; Wright et al., 2005), soil properties (Maire et al., 2015; Simpson et al., 2016), and metal(loid)s concentrations (Chen et al., 2023; Maisto et al., 2013).

For instance, leaf area (LA) plays an important role in environmental adaptation and plant growth, particularly in terms of photosynthetic capacity, energy balance regulation, and ecosystem productivity. In general, a plant's LA is reduced under conditions of drought, high temperature, or high levels of metal(loid)s (Ambo‐Rappe et al., 2011). Leaf thickness (LT) is related to nutrient storage, with thicker leaves containing more palisade tissue, which benefits photosynthetic efficiency and storage capacity (Liu et al., 2020). Furthermore, thicker leaves can store more water to cope with harsh environmental conditions (Zhang, Zhang, et al., 2015). Specific leaf area (SLA) is a critical trait closely associated with plant growth rates, reproductive strategies, and lifespan (Wright & Westoby, 2000). Species with high SLA adopt a more “disposable” strategy, investing less dry matter per leaf, growing quickly, and having shorter leaf lifespans. Conversely, in dry climates and nutrient‐deficient soils, species tend to have lower SLA, enabling them to maintain leaf function under unfavorable conditions for leaf production, thus supporting plant life activities (Dwyer et al., 2014). SLA represents resource capture efficiency. The carbon (C) content of plants reflects C fixation capacity, while phosphorus (P) and nitrogen (N) concentrations in plants are highly correlated across species worldwide (Kerkhoff et al., 2006). Moreover, plant C:N ratio, nutrient concentrations, and mass‐based respiration rates are correlated (Liu et al., 2010). Therefore, studying leaf functional traits aids in explaining and understanding how plants respond to environmental stress.

Species that thrive in mining sites demonstrate remarkable adaptations to disturbed environments and possess the potential for metal(loid)s accumulation within these ecosystems (Chen et al., 2022; Kompała‐Bąba et al., 2020; Zheng et al., 2019). This capability strongly depends on their adaptive traits (Yuan et al., 2023). In such environments, metal(loid)s in the soil often suppress plant height and LA (Delhaye et al., 2016). Lange et al. (2017) observed that plant height and leaf size were lower under metalliferous conditions, specifically with the addition of cobalt (Co). Similarly, elevated metal(loid) levels have been shown to decrease LA and SLA, reducing light absorption and ultimately leading to a decline in the photosynthetic rate (Maisto et al., 2013). However, Lange et al. (2017) found no significant changes in plant SLA between non‐metalliferous and metalliferous soils, potentially due to the varied niches of species and the diversity of metal(loid) types. This suggests that the response of SLA to metal(loid)s stress may be inconsistent. Moreover, shifts in functional traits due to environmental changes can lead to corresponding alterations in stoichiometry ratios (Meunier et al., 2017). For example, under copper (Cu) stress, the C:N:P stoichiometry of *Salix integra* was significantly altered (Cao et al., 2022). A recent study reported that the leaf C:N and C:P ratios of *Rosa chinensis* seedlings were higher than those in the control under the lead (Pb) stress (He et al., 2023). These findings indicate that the impact of metal(loid)s on plant stoichiometric balance warrants further attention (Pedas et al., 2011; Zhang et al., 2023). However, the relationship between elemental and metal(loid) concentrations in plants is highly complex and requires further exploration (Saini et al., 2021; Trethowan et al., 2021). Understanding these relationships is crucial for assessing the ecological effects of mining activities and for developing strategies to mitigate their negative impacts on plant communities.

Here, we investigated the natural restoration of vegetation in the abandoned antimony (Sb) mining sites in Qinglong, Guizhou Province, China. This site provided an ideal setting to study plant functional traits in response to metal(loid) pollution, owing to the long‐term natural succession that has restored the growth of plants resistant to metal(loid)s stress. In the naturally restored Sb slag heap, the predominant herbs include *Artemisia argyi*, *Miscanthus sinensis*, *Ficus tikoua*, *Ageratina adenophora*, etc. (Du et al., 2023). Based on previous findings (Du et al., 2023), we selected and examined the functional traits of these four dominant herb species (*A. argyi*, *M. sinensis*, *F. tikoua*, and *A. adenophora*). Our research explored variations in plant functional traits and investigated the relationship between metal(loid) concentrations and these traits in the dominant species. We hypothesize that: (1) Plants under metal(loid) stress may adopt conservative strategies, such as reducing plant height and leaf area, to maximize environmental adaptation; (2) plant metal(loid) concentrations are jointly regulated by plant elemental concentrations, stoichiometry, and soil properties. The findings of this study aim to enhance our understanding of plant community succession dynamics during the restoration of degraded ecosystems following mining activities. Additionally, the results provide valuable insights into predicting the plant community succession in ecosystems undergoing restoration after mining activities.

## 2 MATERIALS AND METHODS

### 2.1 Site descriptions

**FIGURE 1:**
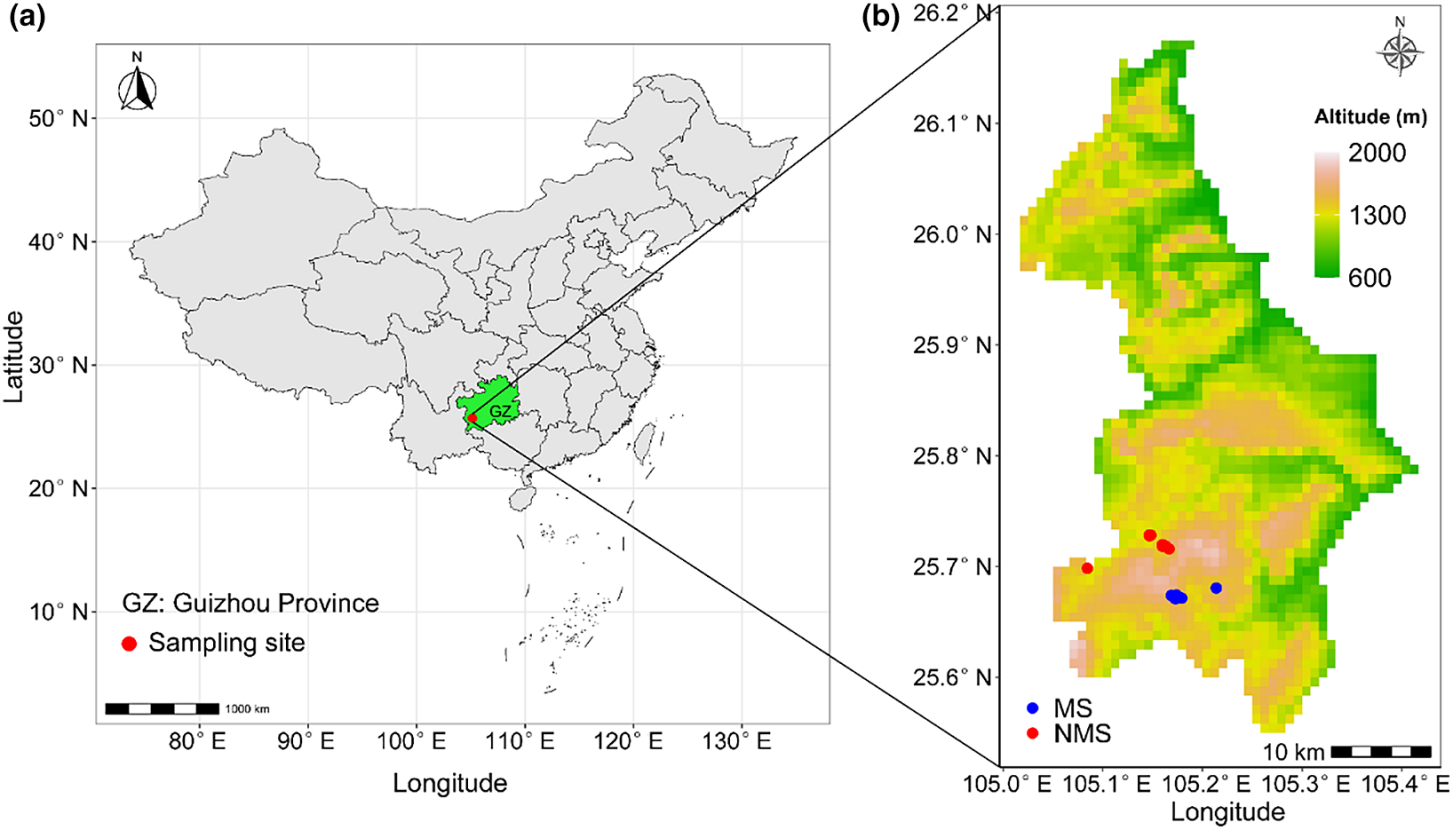
Sampling collection sites. (a) China map and the study area, (b) sampling sites in mining sites (MS) and non‐mining sites (NMS). Blue is mining sites (MS), red is non‐mining sites (NMS).

This study was conducted in Dachang Town, Qinglong County, Guizhou Province, China (25°33′ ~ 26°20′ N, 105°05′ ~ 105°48′ E) (Figure 1). The area exemplifies a typical karst landscape, featuring riverbeds, underground karst caves, sinkholes, and karst gullies. It experiences a subtropical monsoon climate with an average annual temperature ranging from 14.0 to 14.7°C. Annual precipitation averages approximately 1500 mm, mainly from May to October, accounting for over 80% of the year's total. The region enjoys approximately 350 frost‐free days per year and receives between 1454 and 1714 h of sunshine annually. The soil type is mainly brownish‐yellow lime soil. The dominant vegetations are subtropical evergreen and broad‐leaved mixed forests, with dominant tree species including *Betula luminifera*, *Rhus chinensis*, *Vernicia fordii*, *Populus adenopoda*, and *Litsea cubeba*. The shrub species mainly are *Viburnum foetidum*, *Amorpha fruticosa*, *Rubus parvifolius*, and *Rubus coreanus*. Key herbaceous plants include *A. argyi*, *M. sinensis*, *Pteridium aquilinum*, and *A. adenophora* (Du et al., 2023). The Sb slag sites consist of heaps formed from over 50 years of processing residues, releasing metal(loid)s into the environment and impairing surface vegetation (Guo et al., 2023). The current herbaceous vegetation naturally reestablished following the slag dump's abandonment, with no human interventions. Soil analysis details were provided in Table 1, and vegetation composition could be found in Du et al. (2023).

**TABLE 1:** Soil properties and the stoichiometric at mining sites (MS) and non‐mining sites (NMS) with mixed‐effects models.

Variable	Mining sites (MS)	Non‐mining sites (NMS)	*t/z* value	*p* value
pH	5.81 ± 0.27	5.71 ± 0.12	0.18	.86
Soil water content (SWC, %)	22.26 ± 1.74	35.08 ± 2.66	−2.78	.05
Soil organic matter (SOM, g kg^−1^)	29.76 ± 5.18	60.82 ± 5.46	−2.52	.07
Total nitrogen (TN, g kg^−1^)	1.11 ± 0.12	3.45 ± 0.27	**−4.35**	**<.05**
Total phosphorus (TP, g kg^−1^)	2.19 ± 0.36	0.85 ± 0.14	**2.58**	**<.001**
Total potassium (TK, g kg^−1^)	7.43 ± 1.66	2.63 ± 0.45	**2.08**	**<.05**
Available nitrogen (AN, mg kg^−1^)	40.45 ± 5.22	199.36 ± 15.06	**−7.74**	**<.01**
Available phosphorus (AP, mg kg^−1^)	2.70 ± 0.85	0.77 ± 0.01	1.71	.09
Available potassium (AK, mg kg^−1^)	183.96 ± 75.37	70.64 ± 6.19	1.40	.16
Total calcium (TCa, mg kg^−1^)	18.07 ± 4.50	4.18 ± 0.68	1.90	.06
Total antimony (TSb, mg kg^−1^)	15,068.40 ± 3755.27	369.06 ± 94.28	**2.33**	**<.05**
Total arsenic (TAs, mg kg^−1^)	1265.13 ± 548.56	236.29 ± 45.24	1.41	.23
Available antimony (ASb, mg kg^−1^)	70.27 ± 23.89	0.88 ± 0.03	**2.83**	**<.01**
Available arsenic (AAs, mg kg^−1^)	103.54 ± 41.75	6.55 ± 1.20	**3.50**	**<.001**
Exchangeable calcium (ECa, mg kg^−1^)	1110.13 ± 253.71	908.26 ± 179.72	0.38	.72
C:N ratio	28.34 ± 4.51	17.77 ± 1.14	1.33	.25
C:P ratio	15.74 ± 2.94	83.96 ± 11.90	**−4.34**	**<.05**
C:Ca ratio	2.51 ± 0.56	17.62 ± 3.17	−2.48	.07
N:P ratio	0.55 ± 0.06	4.69 ± 0.55	**−5.15**	**<.01**
N:Ca ratio	0.04 ± 0.002	0.28 ± 0.27	−2.43	.07
P:Ca ratio	0.09 ± 0.07	0.07 ± 0.05	−0.03	.98

*Note*: Values are means ± SE. The *t/z* value and *p* value of the mixed‐effect model results are given, highlighting in bold the significant effects (*p* < .05). C:Ca, soil organic matter concentration:soil total calcium concentration; C:N, soil organic matter concentration:soil total nitrogen concentration; C:P, soil organic matter concentration:soil total phosphorus concentration; N:Ca, soil total nitrogen concentration:soil total calcium concentration; N:P, soil total nitrogen concentration:soil total phosphorus concentration; P:Ca, soil total phosphorus concentration:soil total calcium concentration.

### 2.2 Sampling design

Based on our field survey result, four herb species, *A. argyi*, *M. sinensis*, *F. tikoua*, and *A. adenophora*, were selected for this study. These species dominated both in the natural sites (NMS) and Sb mining sites (MS), which provides favorable conditions for our study. *Artemisia argyi* is a perennial plant used traditionally for medicinal and culinary (Yang et al., 2024). *Miscanthus sinensis*, a perennial C_4_ grass, thrives on poor land and boasts advantages of energy capacity. It exhibits the widest natural distribution and superior environmental adaptability compared to other *Miscanthus* species (Wang et al., 2021). *Ficus tikoua*, commonly known as “diguo” in China, is a creeping perennial woody vine belonging to the *Ficus* within the Moraceae. It's native to southern China, India, Vietnam, and Laos, which thrives in diverse environments such as roadsides, riversides, sandy hillsides, wastelands, rock crevices, and open woodlands (Li et al., 2024). *Ageratina adenophora*, also known as Crofton weed, is considered one of the most severe invasive species in Asia, Africa, and Oceania (Tang et al., 2019). It has become the dominant species in many local ecosystems, severely reducing biodiversity and disrupting the composition and normal functions of native plant communities (Tian et al., 2007).

### 2.3 Sampling and trait measurement

To test our hypothesis, 18 10 × 10 m plots were established in early September 2021, including nine plots in mining sites (MS) and nine plots in non‐mining sites (NMS). These plots were used to examine the adaptation strategies of the four dominant herbaceous species (*A. argyi*, *M. sinensis*, *F. tikoua*, and *A. adenophora*) to the mining environment. Within each plot, 5 to 15 individuals of each species were randomly sampled. We selected mature leaves that were fully unfolded and sun‐exposed, ensuring they were representative of the plant's natural state and minimally affected by developmental phases or external damage, such as animal activity (Liu et al., 2023).

Firstly, we measured the height of the selected plants (Wang et al., 2022). Immediately after, we collected complete plant specimens, including both aboveground and underground parts, as well as leaf samples. These were immediately transported to the laboratory for further analysis. In the laboratory, we recorded the leaf area (LA) using a plant leaf scanning analysis system (WinRHIZO, AgriPheno, Canada) and measured leaf thickness (LT) with electronic vernier calipers, achieving an accuracy of 0.01 mm. Subsequently, the leaves were dried at 75°C until reaching a constant mass, and their weight was recorded to calculate the specific leaf area (SLA). The SLA was defined as the ratio of LA to dry leaf mass (Pérez‐Harguindeguy et al., 2013; Wang et al., 2022). For elemental analysis, the plant samples were ground into a fine powder. The concentrations of carbon (C) and nitrogen (N) were determined using an element analyzer (Vario Max cube, Elementar, Germany). To measure the concentrations of phosphorus (P), calcium (Ca), antimony (Sb), and arsenic (As), 0.15 g of the powdered samples was digested with 6 mL HNO_3_ at 120°C for 45 min, followed by an additional digestion with 1 mL H_2_O_2_ for 30 min in a hot block system (ED36, Lab Tech, China). Finally, the concentrations of Sb, As, P, and Ca in the digestion solution were determined using inductively coupled plasma optical emission spectrometry (ICP‐OES) (iCAP7400, Thermo Scientific, USA).

### 2.4 Soil collection and determination

Three mixed soil samples were randomly collected within each 10 × 10 m plot. Soil samples were taken from the surface (0–10 cm depth), for a total of 54 soil samples. Each sample was divided into two parts. The first part was oven‐dried at 105°C until a stable weight was reached to determine soil water content (SWC) (Xing et al., 2024). The second part was air‐dried, crushed, and sieved through 2 mm and 0.149 mesh sieves to standardize the soil particle size. This processed soil was used for measuring soil properties and metal(loid) concentrations (Table 1).

Soil pH was measured using a pH meter (PHS3C, Leici Instruments, China) with a soil‐to‐distilled water ratio of 1:2.5 (*w*/*v*). Soil organic matter (SOM) content was quantified employing the dichromate oxidation method (Nelson & Sommers, 1996). Total nitrogen (TN) content was measured using an elemental analyzer (Vario MAX cube, Elementar, Germany). Total phosphorus (TP) content was measured by alkali fusion‐Mo‐Sb anti‐spectrophotometric as presented by the Ministry of Environmental Protection of China (HJ 632–2011) (Liu et al., 2022). Total potassium (TK) content was digested in a nickel crucible with sodium hydroxide at 450°C. Available nitrogen (AN) was assessed using the alkaline hydrolysis distillation method (Bao, 2000). Available phosphorus (AP) was analyzed using hydrochloric acid and sulfuric acid solution distillation method (LY/T1232‐1999). Available potassium (AK) was extracted using 1 mol L^−1^ ammonium acetate and then measured using flame photometry (Bao, 2000). Exchangeable calcium (ECa) was measured utilizing an atomic absorption spectrophotometer (ICE 3500; Thermo Scientific, United States).

Furthermore, the soil samples were passed through a 0.149 mm strainer for Ca and metal(loid) concentrations analysis. Specifically, an accurately weighed 0.1 g soil sample was digested in a glass tube with a mixture of HNO_3_ and HCl mixture (1:3 *v*/*v*) using a hot block system (ED36, Lab Tech, USA) and kept at a slight boil for 2 h. Then, the concentrations of Ca, Sb, and As were determined using inductively coupled plasma mass spectrometry (7700, Agilent, United States) (Xing et al., 2023). For the determination of available antimony (ASb) and arsenic (AAs), a 1 M NH_4_H_2_PO_4_ solution was used for extraction (Zhong et al., 2020).

### 2.5 Calculation and statistical analysis

The data analysis and graphical plotting were completed using R (version 4.2.0) (R Core Team, 2022). To facilitate the interspecies comparison of differences in mining environment, we employed log response ratios (Ln*RR*) as a metric to quantify the effect sizes of impacts on the four species (Deraison et al., 2015; Hedges et al., 1999; Jiang et al., 2023). To enable robust significance testing, we modified the Ln*RR* calculation formula as follows:

$$\mathrm{Ln}\mathrm{RR}=\ln\left(\frac{\mathrm{Xt}}{\bar{\mathrm{Xc}}}\right)=\ln\left(\mathrm{Xt}\right)-\ln\left(\bar{\mathrm{Xc}}\right)$$

where Xt represents the values of response variables in the plot at the MS, and $\bar{Xc}$ denotes the mean value of response variables across all plots at the NMS.

Before performing statistical analysis, the residual variables were tested for normality using the Shapiro–Wilk test, and data were log‐transformed as necessary to meet the assumptions of the analysis. To assess the differences between MS and NMS, we utilized a liner mixed‐effects model or a generalized linear mixed‐effects model with a Poisson distribution, considering soil properties (pH, SWC, SOM, etc.) and metal(loid)s concentrations (TSb, TAs, ASb, and AAs) as predictors. Furthermore, a linear mixed‐effects model was utilized to test the interaction effects between sites and species on plant ecological stoichiometry. The Tukey method was used to correct for multiple comparisons, incorporating the plot as a random factor as determined by the Akaike's Information Criterion (AIC) value.

A two‐way ANOVA was used to examine the effects of the four species, sites, and their interactions on plant morphological traits. Furthermore, one‐way ANOVA and Tukey method test (*p* < .05) were employed to evaluate significant differences in plant height, LA, LT, and SLA among the four species. When the data did not conform to a normal distribution, the *wilcox.test* function and *dunnTest* function were used for non‐parametric testing, utilizing the dunn test from the “FSA” package (Ogle et al., 2022). To explore the relationship between plant metal(loid) concentrations and ecological stoichiometry, a linear mixed‐effects model was employed, incorporating plot and species as random factors.

To estimate and test the effects of predictors, we employed a linear mixed‐effects model to construct a piecewise structural equation model (SEM) using the “piecewiseSEM” package (Lefcheck, 2016). SEMs enabled the integration of multiple response and predictor variables into a comprehensive model, facilitating a deeper understanding of the relationships between predictors and responses (Lefcheck, 2016; Pearl, 2012). In the models, plot and species were included as random factors in our model. Fisher's *C* statistics were calculated for all models, where a *p* value >.05 indicated a good fit of the data. The lowest AIC was used to evaluate the model (Shipley, 2009). Following the initial model fitting, we iteratively removed non‐significant interactions to arrive at the optimal model (Grace & Bollen, 2005). For each dependent variable, we calculated the explained variability as the marginal *R* ^2^ value (fixed effect) and conditional *R* ^2^ value (both fixed and random effects) (Nakagawa et al., 2017). All the models were carried out using the *lmer* and *glmmTMB* functions in the “lme4,” “lmerTest,” and “glmmTMB” packages (Bates et al., 2015; Brooks et al., 2017).

We used the first (PC1), second (PC2), or third (PC3) axis of principal component analysis (PCA) to address the high correlation among multiple environmental variables (Figures S1 and S8, Tables S1–S5) (Jiang et al., 2023). The combined explanation of PC1, PC2, and PC3 accounted for between 73.55% and 98.17% of the variance (Figure S8, Tables S1–S5). We classified the variables into five categories based on the properties prior to implementing SEMs (Jiang et al., 2023). These categories were: (1) soil physicochemical properties, that is, pH, SWC, SOM, TN, TP, TK, TCa, AN, AK, and ECa; (2) soil metal(loid) concentrations, including TSb, TAs, ASb, and AAs; (3) plant morphological traits such as plant height, LT, LA, and SLA; (4) plant elemental composition, including C, N, P, and Ca; (5) element ratios in plants, including C:N, C:P, C:Ca, N:P, N:Ca, and P:Ca. An a priori model was used to validate the reasonableness of the current model (Figure S2). The PCA was constructed using the *PCA* function in the “FactoMineR” package (Sebastien et al., 2008).

Finally, the importance of variables, including soil properties, soil metal(loid) concentrations, and all plant traits, on plant metal(loid) concentrations was determined using “randomForest” and “rfPermute” packages (Archer, 2023; Liaw & Wiener, 2002). All figures were plotted using the “ggplot2” and “ggpubr” packages (Alboukadel, 2020; Wickham, 2016).

## 3 RESULTS

### 3.1 Soil properties and plant morphological traits

The soil TP, TK, TSb, ASb, and AAs in the MS were significantly higher compared to those in NMS (*p* < .05), whereas the TN, C:P, and N:P ratios of the MS were significantly lower than those in the NMS (*p* < .01) (Table 1). Additionally, SWC, SOM, C:Ca, and N:Ca ratios decreased in MS compared to NMS, while AP and TCa increased (*p* < .1) (Table 1).

**FIGURE 2:**
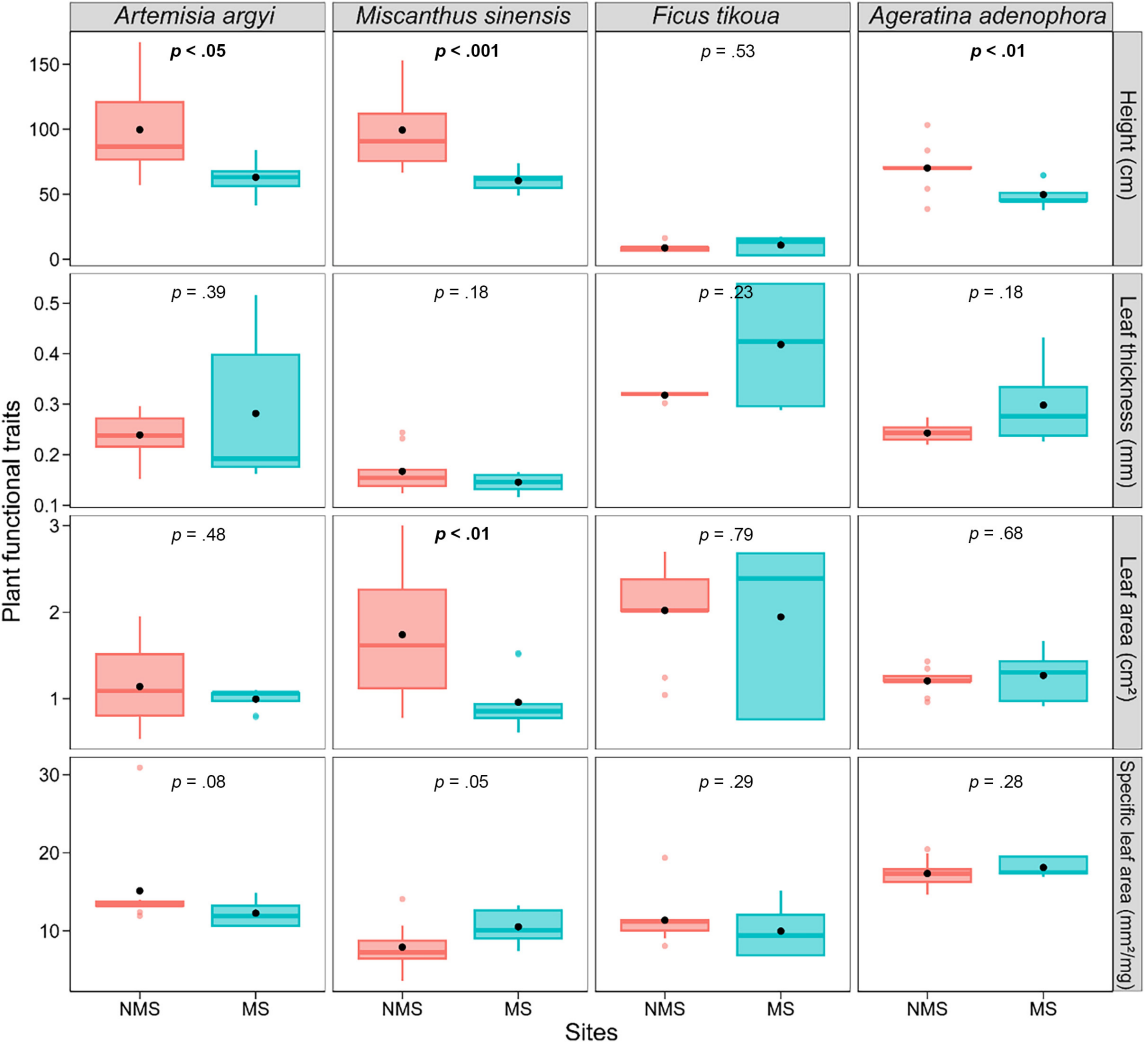
Plant natural height, leaf thickness, leaf area, and specific leaf area of four species at mining site (MS) and non‐mining site (NMS). The *p* value of the ANOVA results is given, highlighting in bold the significant effects (*p* < .05).

**FIGURE 3:**
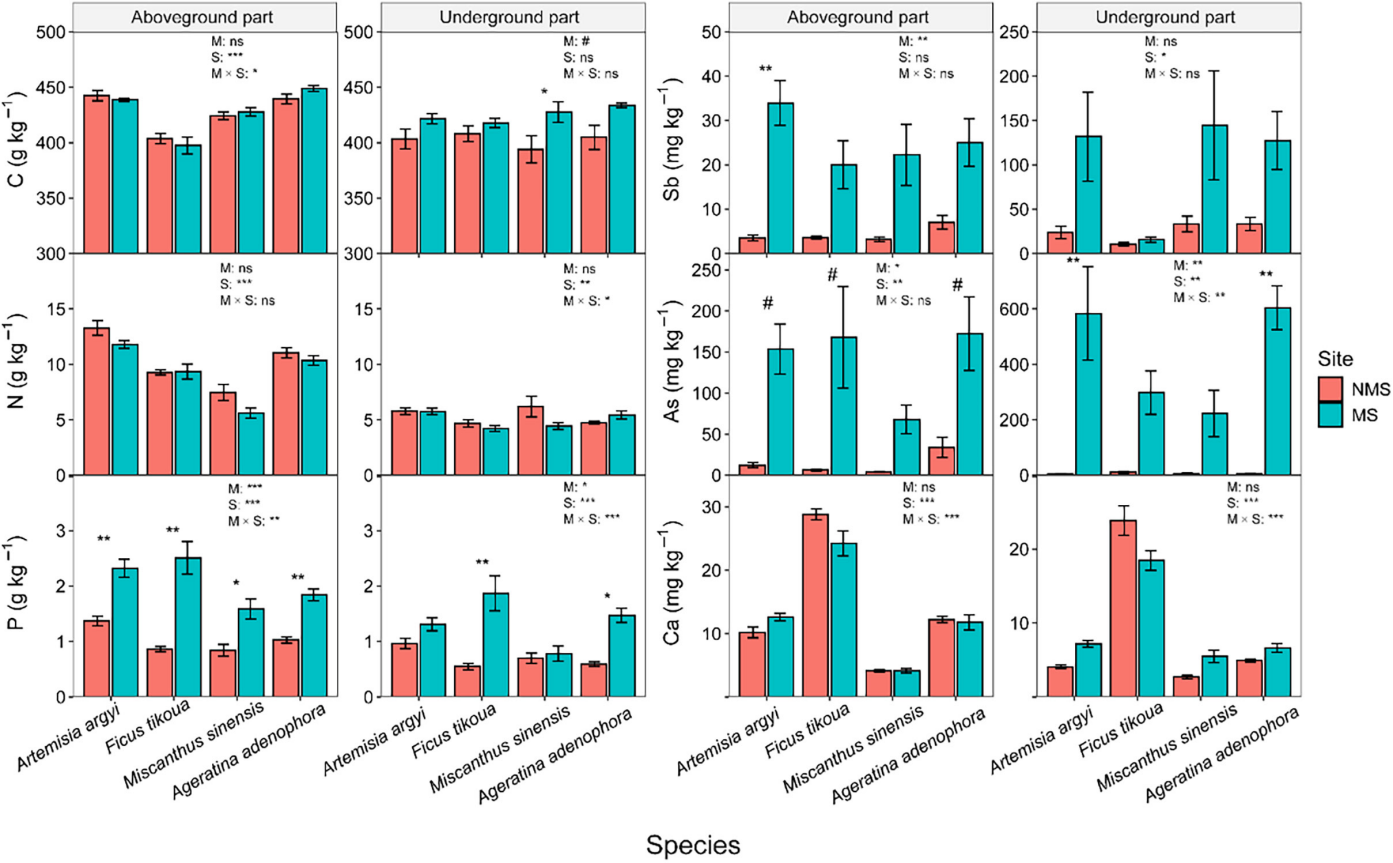
The carbon (C), nitrogen (N), phosphorus (P), antimony (Sb), arsenic (As), and calcium (Ca) concentrations in different plant organs of four species (*Artemisia argyi*, *Miscanthus sinensis*, *Ficus tikoua*, and *Ageratina adenophora*) at mining sites (MS) and non‐mining sites (NMS). M are the sites, including MS and NMS; S are the species, including *Artemisia argyi*, *Miscanthus sinensis*, *Ficus tikoua*, and *Ageratina adenophora*. ns represents *p* > .1, ^#^Represents the *p* < .1, *Represents *p* < .05, **Represents *p* < .01, ***Represents *p* < .001.

Significant differences in plant height, LT, LA, and SLA among different sites and species were found (Figure 2, Table 2). Specifically, the heights of *A. argyi*, *M. sinensis*, and *A. adenophora* in MS were significantly lower than those in the NMS. *M. sinensis* exhibited significantly smaller LA in MS than in NMS (*p* < .01) (Figure 2). Notably, *F. tikoua* exhibited the thickest LT and the biggest LA in MS among the four species, whereas *A. adenophora* exhibited higher SLA (Figure 2). The C, N, and Ca concentrations of the four species in MS were similar to those in NMS (*p* > .05) (Figure 3).

**TABLE 2:** Results of ANOVA or non‐parametric testing the impacts of sites (MS and NMS), species (*Artemisia argyi*, *Miscanthus sinensis*, *Ficus tikoua*, and *Ageratina adenophora*), and their interactions on plant natural height, leaf thickness, leaf area, and specific leaf area.

Factors	Height			Leaf thickness			Leaf area			Specific leaf area		
	df	*H* value	*p* value	df	*H* value	*p* value	df	*H* value	*p* value	df	*H* value	*p* value
Sites	1	**9.18**	**<.01**	1	0.82	.364	1	**4.39**	**<.05**	1	0.00	.964
Species	3	**43.60**	**<.001**	3	**45.08**	**<.001**	3	**13.53**	**<.01**	3	**44.80**	**<.001**
Sites × Species	3	3.90	.273	3	1.73	.630	3	5.05	.168	3	2.84	.416

*Note*: The df, *F* (*H*), and *p* values are given, highlighting in bold the significant effects (*p* < .05).

### 3.2 Nutrient elements, ratios, and metal(loid)s in plant tissues

The P concentrations in the aboveground parts of the four species in MS were significantly increased compared to NMS (*p* < .05) (Figure 3, Figure S3). The Sb and As concentrations in *A. argyi* and the As concentrations in *F. tikoua* and *A. adenophora* in MS were increased compared to NMS (*p* < .1) (Figure 3). The Sb concentration in the aboveground parts of *A. argyi* was significantly higher than in *A. adenophora* (*p* < .05) (Figure S3). The C, N, and Ca concentrations of the four species in MS were similar to those in NMS (*p* > .05) (Figure 3). Contrarily, the As concentrations in the underground parts of the four species were higher compared to their aboveground parts, and the As concentrations in the underground parts of *A. argyi* and *M. sinensis* were significantly higher than those of *F. tikoua* (*p* < .05) (Figure S3). Notably, plant Sb, As, and Ca concentrations were affected by the interactions between sites and species (*p* < .05) (Figure 3).

**FIGURE 4:**
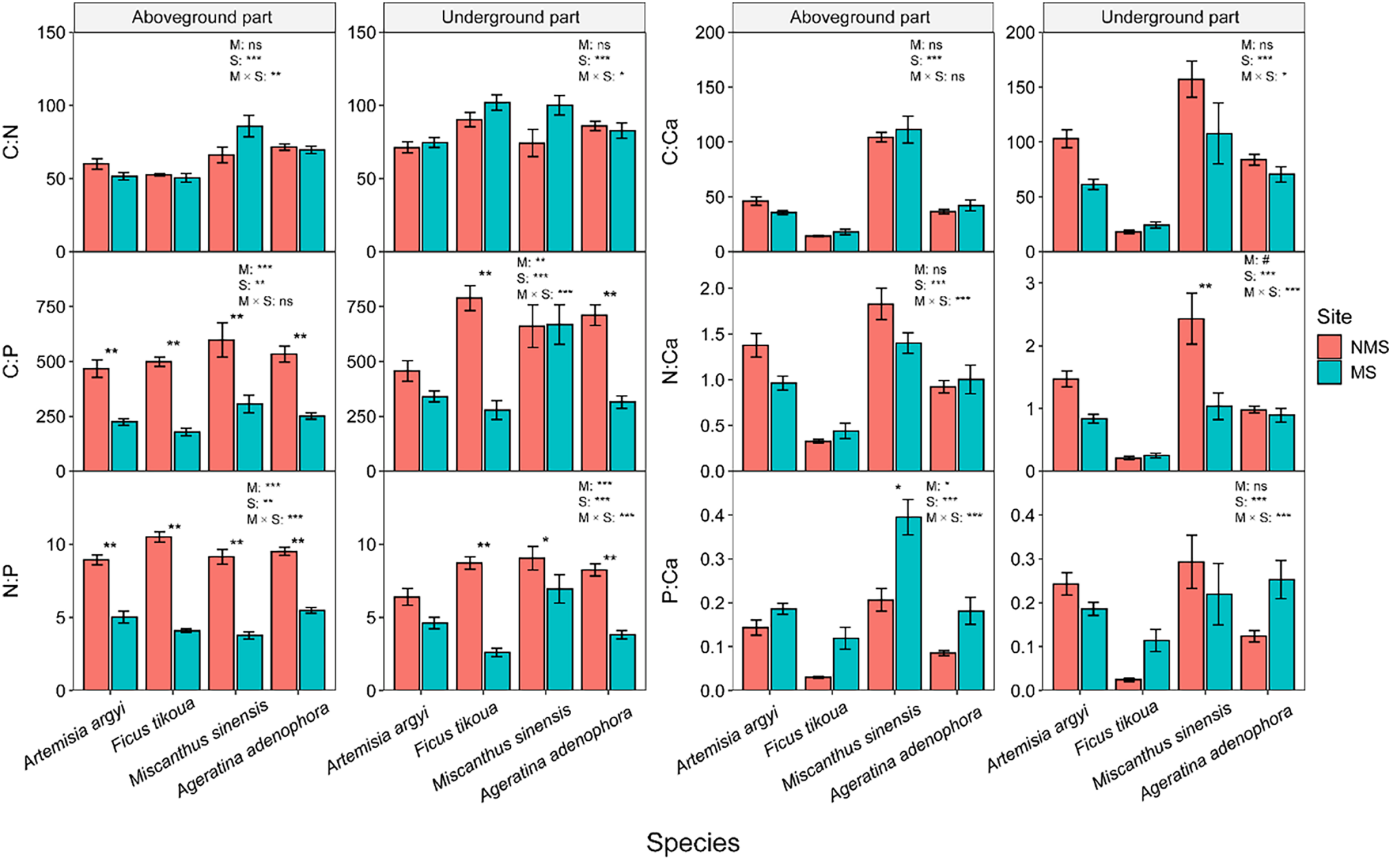
The C, N, P, and Ca element ratios in different plant organs of four species (*Artemisia argyi*, *Miscanthus sinensis*, *Ficus tikoua*, and *Ageratina adenophora*) at mining sites (MS) and non‐mining sites (NMS). M are the sites, including MS and NMS; S are the species, including *Artemisia argyi*, *Miscanthus sinensis*, *Ficus tikoua*, and *Ageratina adenophora*. ns represents *p* > .1, #Represents the *p* < .1, *Represents *p* < .05, **Represents *p* < .01, ***Represents *p* < .001.

Compared to NMS, the N:P ratios of the four species were significantly decreased (*p* < .01) (Figure 4). The C:P ratio in aboveground parts of the four species decreased significantly, while the P:Ca ratio in underground parts increased significantly (*p* < .05) (Figure 4). There were significant differences among different species (*p* < .01) (Figure 4). The C:N ratio of *M. sinensis* was significantly higher than those of *A. argyi*, *F. tikoua*, and *A. adenophora* (*p* < .05) (Figure S3). The N:P ratios of *A. argyi* and *M. sinensis* were significantly higher than those of *F. tikoua* (*p* < .05) (Figure S3). The N:Ca and P:Ca ratios of *F. tikoua* were significantly higher than other species (*p* < .05) (Figure S3). In addition, differences in plant element ratios, except for the C:Ca ratio in aboveground parts, were affected by both plot and species (*p* < .05) (Figure 4).

### 3.3 Relationship between elements, their ratios, and metal(loid)s in plant

**FIGURE 5:**
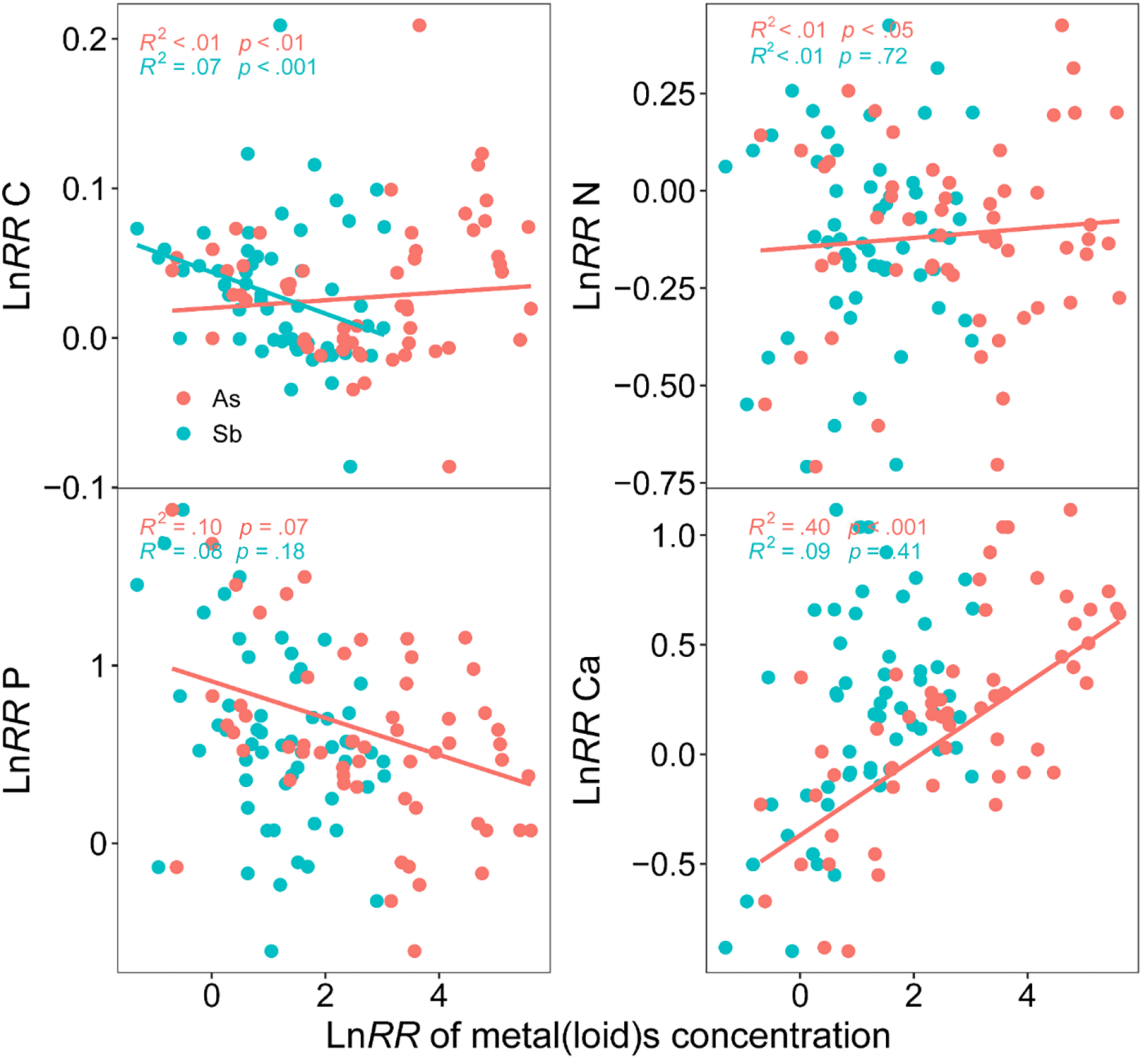
Linear mixed‐effect models showing the relationship between ratios of C, N, P, and Ca and metals As and Sb in plants. The fixed *R* ^2^ and model *p* values are given in the figure. The red point represents the Ln*RR* of plant As concentration, the white point represents the Ln*RR* of plant Sb concentration. *x*‐axis is the Ln*RR* of plant As and Sb concentrations, *y*‐axis is the Ln*RR* of plant C, N, P, and Ca concentrations.

The plant Sb concentration was significantly negative related to C concentration, while As concentration was significantly positive related to C, N, and Ca concentrations (*p* < .05) (Figure 5). Sb and As concentrations in *F. tikoua* were negatively related to C, N, and P concentrations (*p* < .01) (Figure S4). As concentration in *A. argyi* was significantly positive correlated with C and Ca concentrations (*p* < .01), whereas it was negatively correlated with P concentration (*p* < .001) (Figure S4). There were significantly positive relationships between As and C, N, P, and Ca concentrations in *A. adenophora* (*p* < .001) (Figure S4).

**FIGURE 6:**
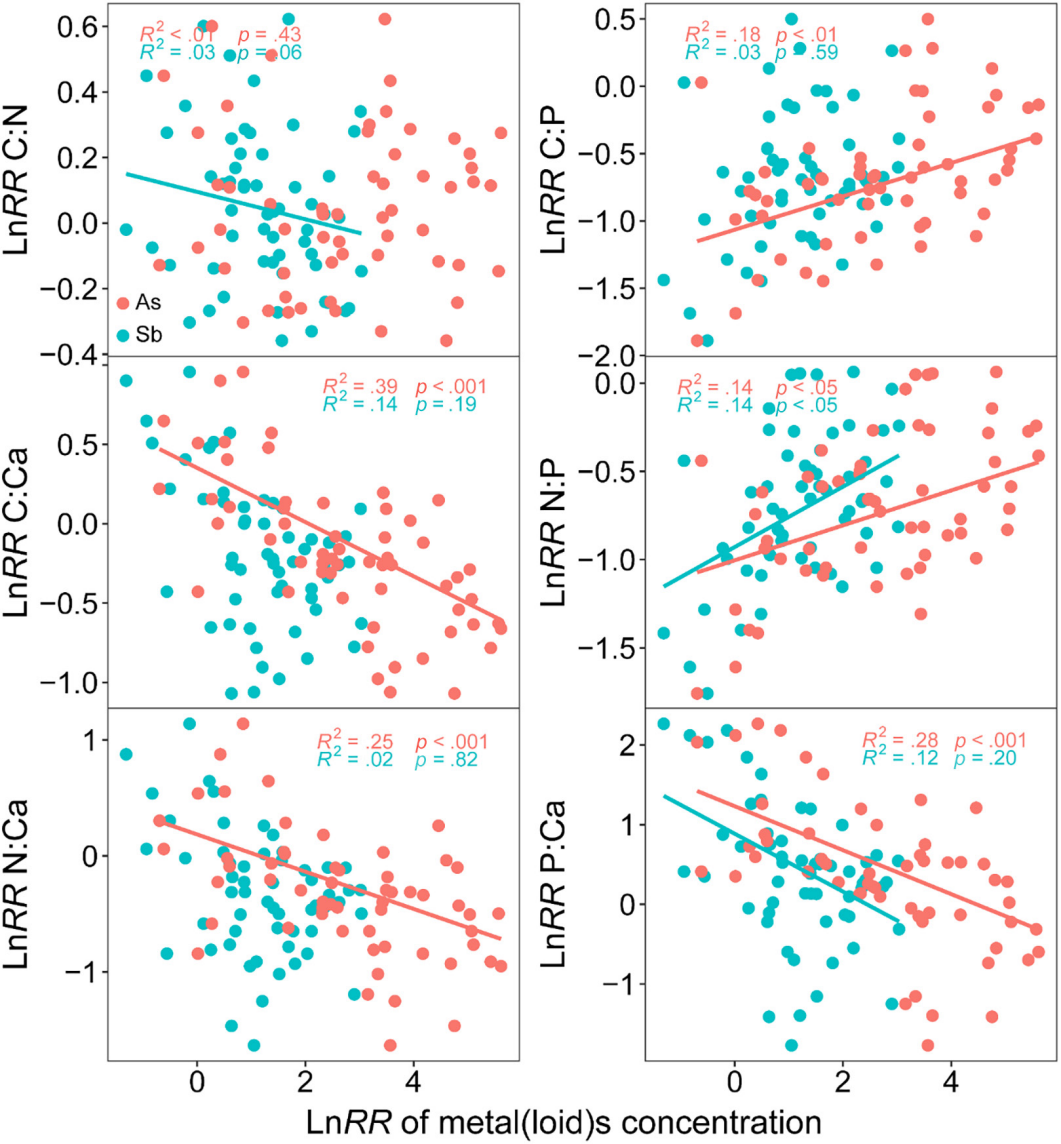
Linear mixed‐effect models showing the relationship between ratios of C:N, C:P, C:Ca, N:P, N:Ca, and P:Ca and metals As and Sb in plants. The fixed *R* ^2^ and model *p* values are given in the figure. The red point represents the Ln*RR* of plant As concentration, the white point represents the Ln*RR* of plant Sb concentration. *x*‐axis is the Ln*RR* of plant As and Sb concentrations, *y*‐axis is the Ln*RR* of plant ratios of C:N, C:P, C:Ca, N:P, N:Ca, and P:Ca in plants.

There was a significantly negative correlation between Sb concentration and the P:Ca ratio in plants, whereas there was a significantly positive correlation between Sb concentration and the N:P ratio (*p* < .05) (Figure 6). Plant As concentration was significantly positive correlated with the C:P and N:P ratios, while it was significantly negative correlated with the C:Ca, N:Ca, and P:Ca ratios (*p* < .05) (Figure 6). Sb concentration in *F. tikoua* was significantly negative related to the C:Ca, N:Ca, and P:Ca ratios, while it was significantly positive related to the C:N, C:P, and N:P ratios (*p* < .01) (Figure S5). As concentration in *A. argyi* was significantly negative correlated with the C:Ca, N:Ca, and P:Ca ratios, while it was significantly negative correlated with C:N and C:P ratios (*p* < .01) (Figure S5). Plant As concentration in *A. adenophora* was significantly negative correlated with the C:Ca and N:P ratios (*p* < .05) (Figure S5). Additionally, the relationship between Sb and As concentrations in the plant of the four species was significantly positive (*p* < .05) (Figures S6 and S7).

### 3.4 Pathways determining As and Sb concentrations in plant

**FIGURE 7:**
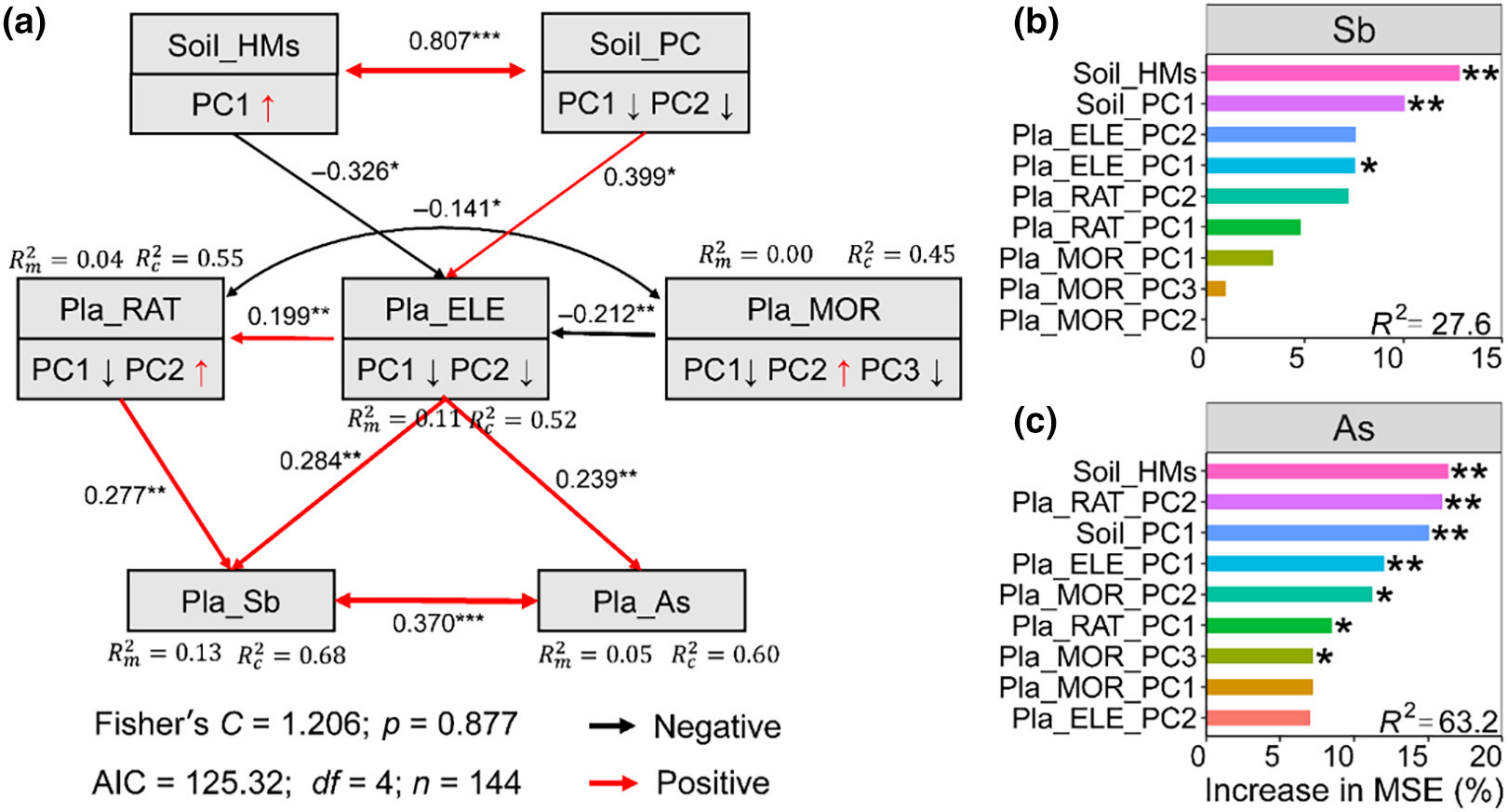
Piecewise structural equation models (SEM) revealing the direct and indirect effects on the plant Sb and As (a) concentration; significant predictors of plant Sb (b) and As (c) concentrations by random forest modeling analysis. Black and red solid arrows indicate positive and negative associations, respectively (*p* < .05). Dotted lines represent pathways that are not significant. Numbers adjoining the arrows indicate significant standardized path coefficients. The arrow width is proportional to the strength of the association. Fisher's *C* statistic, *p*‐value, and Akaike information criterion (AIC) of each SEM are presented under the model. The amount of variance explained by the model (*R* ^2^) is shown for the response variables. Asterisks indicate statistical significance (***, *p* < .001; **, *p* < .01; *, *p* < .05). Soil_HMs, soil metals concentration; Soil_PC, soil physical and chemical properties; Pla_ELE, plant C, N, P, Ca element; Pla_RAT, plant C, N, P, Ca ratios; Plan_MOR, plant height, plant leaf area, plant thickness, and specific leaf area; Pla_Sb, plant Sb concentration; Pla_As, plant As concentration.

Soil metal(loid)s and other properties had direct effects on plant Sb and As concentrations (*p* < .05) (Figure 7a). Plant element traits had direct effects on element ratios and increased plant Sb concentration (*p* < .01). Positive direct effects of plant element concentrations and ratios were observed on plant Sb and As concentrations (*p* < .05) (Figure 7). Random forest modeling results showed that soil metal(loid)s concentrations (ASb and AAs concentrations), PC1 of soil properties (soil TN and AN concentrations), and PC2 of plant element (plant Ca concentration) were important variables affecting plant Sb concentration (*p* < .05) (Figure 7b, Tables S1–S3). Metal(loid)s concentrations (ASb and AAs concentrations), PC2 of plant ratios (C:P and N:P ratios), PC1 of soil properties (soil TN and AN concentrations) were important variables affecting plant As concentration (*p* < .05) (Figure 7c, Tables S1–S3).

## 4 DISCUSSION

### 4.1 Soil properties changes after mining activities

Undoubtedly, the concentrations of Sb and As in soils at the mining sites were geochemically elevated (up to 15,068.4 and 1265.1 mg kg^−1^, respectively), significantly exceeding the background levels in Guizhou province soils (2.24 and 20 mg kg^−1^, respectively) (He et al., 2021) and European soils (0.5 to 30 and 2 to 300 mg kg^−1^, respectively) (Tóth et al., 2016). Consistent with previous studies, mining activities have resulted in a serious excess of mental concentrations (Chen et al., 2023; Xing et al., 2024). High emissions of metal(loid)s have occurred through the disposal of effluents and slag (Huang et al., 2018). Metal(loid)s, resistant to degradation and difficult to migrate, continually accumulate in soil and become permanent pollutants (Zhao et al., 2019). Meanwhile, mining activities significantly decreased the C and N concentrations, consistent with previous studies (Hou et al., 2018; Tian et al., 2022). In the current study, Sb mining significantly destroyed soil structure and lowered fertility, with significantly reduced TN, AN, C:P, and N:P ratios (Table 1). Mining activities declined biodiversity, including vegetation and microorganisms, thereby reducing the input of N and C (Lal, 2003). Moreover, soil environmental destruction hinders soil nutrient recovery, leading to a reduction in soil C and N stocks (Feng et al., 2019; Qin et al., 2020). We found significantly elevated concentrations of TP and TK (Table 1). Mining activities led to an increase in P and Ca content, attributed to the inheritance of the chemical properties of the parent rock by the soil in karst rocky areas (Wei et al., 2018). Changes in soil elements (such as C, N, P, and Ca) inevitably lead to alterations in element ratios. Soil C, N, and P stoichiometry are important indicators for measuring soil nutrient composition and balance (Zhang, Wang, et al., 2015). Mining activities significantly decreased the C:P and N:P ratios, directly related to the increase in P concentration and the decrease in N concentration (Table 1). In addition, the soil C:Ca and N:Ca ratios decreased after mining activities due to the improved soil Ca concentration (Table 1).

### 4.2 Adaptation strategies of dominant species in growth

Functional traits and growth strategies of plants are influenced by soil properties (Maire et al., 2015; Simpson et al., 2016). In the current study, the functional traits of *A. argyi*, *M. sinensis*, and *A. adenophora* shifted toward conservative strategies in the mining environment, exhibiting lower plant height and leaf N concentration, as well as smaller leaf area (Figures 2 and 3, Figure S3). Delhaye et al. (2016) found that plant height and leaf area of most species decreased with increasing soil Cd and Co concentrations. Lower plant height indicates that less N is needed for plant growth, thereby reducing water requirements. Further, these species allocated more energy to defense under metal(loid) stress (Audet & Charest, 2008; Maestri et al., 2010). We found that the LT and SLA of the four species showed no significant changes between the MS and NMS, suggesting that these species did not reduce investment in leaves and maintained photosynthetic capacity and growth compared to the NMS (Garrido et al., 2021).

The P, Sb, and As concentrations in plants from the MS were obviously increased (Figure 4), while the C:P and N:P ratios decreased (Figure 5). Mining activities resulted in N‐limited plant growth in Sb mining sites, with soil P significantly increased under mining activities (Table 1). The C:P ratio (mean 293.17) of the four species in both MS and NMS was higher than the global dataset average (C:P ratio: 232 ± 145) (Elser et al., 2000), suggesting that the four dominant species have a higher P absorption ability. Soil P availability provided the necessary P elements for plant growth (Table 1). The N:P ratio is considered an indicator of plant growth and nutritional status. Our findings showed that the plant N:P ratios in the MS (average: 4.56) were significantly decreased compared to the NMS (average: 9.05). Both MS and NMS ratios were lower than 14, suggesting that plant growth was limited by N concentration. These results explained why plants in the MS have smaller leaves (Figure 2). In addition, mining activities further exacerbated the restriction of plant growth due to soil N concentration (Koerselman & Meuleman, 1996). The ecotoxicological effects of metal(loid)s may also result in significantly reduced absorption of nutrient elements, mainly due to competition for binding sites (Zheng et al., 2018). Metal(loid) concentrations negatively affected the C and N fixation performance of plants, leading to a decrease in plant C:P and N:P ratios.

### 4.3 Driving mechanisms for the plant metal(loid) concentrations

We found that plant C concentration decreased with increasing plant Sb concentration, whereas it increased with plant As concentration (Figure 4). A reasonable explanation is that metal(loid) stress inhibits cellular metabolism, reducing cell growth and proliferation (Ahmad et al., 2021). In addition, metal(loid) stress reduces the photosynthetic efficiency of plants, leading to a decreased plant C fixation (Chai et al., 2017). Chai et al. (2017) found that the growth of *F. tikoua* leaves was significantly inhibited when Sb concentration was higher than 30 μmol Sb L^−1^, which is consistent with our results showing a significantly negative correlation between C and Sb concentrations (Figure S4). The positive relationship between C and As concentrations can be attributed to the fact that As (V) and As (III) are two oxidative forms of As predominant in soil and are more available to plants (Souri et al., 2017). More As is absorbed when plants fix C. Ca as a signal element activating plant defense function plays a regulatory role in plant metal(loid) tolerance (Sanders et al., 2002). It has been reported that exogenous Ca^2+^ application can modulate physiological and biochemical responses to alleviate metal(loid)s stress (de la Torre et al., 2013). In the current study, a significantly positive relationship was found between plant As and Ca concentrations, increasing plant Ca concentration, and resulting in negative correlations with C:Ca, N:Ca, and P:Ca ratios (Figure 4). On the one hand, plants growing in Ca‐rich areas absorbed more Ca than in Ca‐deficient areas. On the other hand, a high‐Ca environment promotes the synthesis of oxalic acid and Ca oxalate in plants, aiding in the detoxification of metal(loid)‐contaminated soil (Bityutskii et al., 2019; Jalilvand et al., 2020). We observed a significantly positive relationship between N:P ratio and Sb and As concentrations (Figure 5). This might be due to the phytotoxicity caused by excessive Sb and As in the soil, which can influence water homeostasis, cellular permeability, and changes in physiological functions (Kamran et al., 2018). However, our results differ from the relationship under Cu stress observed by Cao et al. (2022), which may be related to the complexity of metal(loid) types and the initial soil nutrient content.

We found that the positive effects of plant Sb and As concentrations were mediated through changes in plant element concentrations and ratios (Figure 7). The positive association between plant element ratios and increasing Cu concentration is consistent with the findings of Cao et al. (2022). Meanwhile, we provided evidence for the negative response of plant elements to soil metal(loid)s (Figure 7). Our findings suggest that plant element concentrations and ratios facilitate greater absorption of soil metal(loid)s, thereby increasing plant metal(loid) concentrations at polluted sites. This result complements empirical evidence that plant element ratios increase metal(loid)s concentrations (Cao et al., 2022; Song et al., 2019). Moreover, soil available metal(loid) concentrations (ASb and AAs) negatively affected plant growth and element concentrations (Figures 2, 3, 4), which is consistent with previous results (Song et al., 2019). We found that soil physical and chemical properties have a significantly positive relationship with soil metal(loid) concentrations. Soil properties critically influence the speciation, bioavailability, and solubility of As (Bissen et al., 2003). Previous studies have also suggested that the solubility of As increases with rising pH, indicating that As remains biologically toxic as soil pH increases (Romero‐Freire et al., 2014; Simón et al., 2010; Yang et al., 2002). In addition, plant Sb and As concentrations were driven by plant C, N, and Ca concentrations, consistent with phytoremediation characteristics (Oyuela Leguizamo et al., 2017). In summary, the cycling of chemical elements between plants and soil is a complex process, where nutrient content and stoichiometry play important roles in the relationship with metal(loid) absorption (Cao et al., 2022; Song et al., 2019). Furthermore, the relationship between multiple metal(loid)s and plant stoichiometry is very complex, and the mechanisms still require further study.

## 5 CONCLUSIONS

This study revealed the response of plant functional traits to mining activities and their role in the metal(loid)s accumulation capacity in dominant species. The dominant species tended to adopt conservative strategies (such as lower height, lower C and N concentrations, and higher Ca concentration) to better maintain growth and reproduction. Additionally, plants adjusted metal(loid) concentrations through changes in height and chemical traits. These findings have important implications for understanding and predicting plant community succession dynamics in the restoration of degraded ecosystems following mining activities. The role of function traits in metal(loid)s mining environments should be considered in vegetation restoration efforts.

## AUTHOR CONTRIBUTIONS

**Zhongyu Du:** Conceptualization (equal); data curation (equal); formal analysis (equal); investigation (equal); methodology (equal). **Shufeng Wang:** Writing – review and editing (equal). **Wenli Xing:** Investigation (equal). **Liang Xue:** Conceptualization (equal); investigation (equal). **Jiang Xiao:** Investigation (equal). **Guangcai Chen:** Conceptualization (equal); funding acquisition (equal); project administration (equal).

## CONFLICT OF INTEREST STATEMENT

The authors have no conflict of interest to declare.

## Supporting information


Data S1.

## Data Availability

The data and R code of the study are archived in the GitHub repository: https://github.com/ZhongYu‐Du/PFTs‐Qinglong‐herbaceous‐ECE.
