# Supplementary material for: Plant traits regulated metal(loid)s in dominant herbs in an antimony mining area of the karst zone, China
Source: Ecol Evol. 2024 Aug 23;14(8):e70212. doi: 10.1002/ece3.70212 (PMC11343610; doi:10.1002/ece3.70212)
Supplement: Supplementary file 1 — Data S1. [file ECE3-14-e70212-s001.docx]

**Supplementary Information**

**Plant traits regulated metal(loid)s in dominant herbs in an antimony mining area of the Karst Zone, China**

Zhongyu Du, Shufeng Wang, Wenli Xing, Liang Xue, Jiang Xiao, Guangcai Chen^*^

Research Institute of Subtropical Forestry, Chinese Academy of Forestry, Hangzhou 311400, China.

***Corresponding author**

Prof. Guangcai Chen, Research Institute of Subtropical Forestry, Chinese Academy of Forestry Hangzhou 311400, China.

E-mail: [guangcaichen@sohu.com](mailto:guangcaichen@sohu.com); [gcchen@caf.ac.cn](mailto:gcchen@caf.ac.cn)

**Supplementary Figures:**

**Figure S1** Pairwise correlation matrix for plant traits, soil properties and metal(loid)s concentration.

**
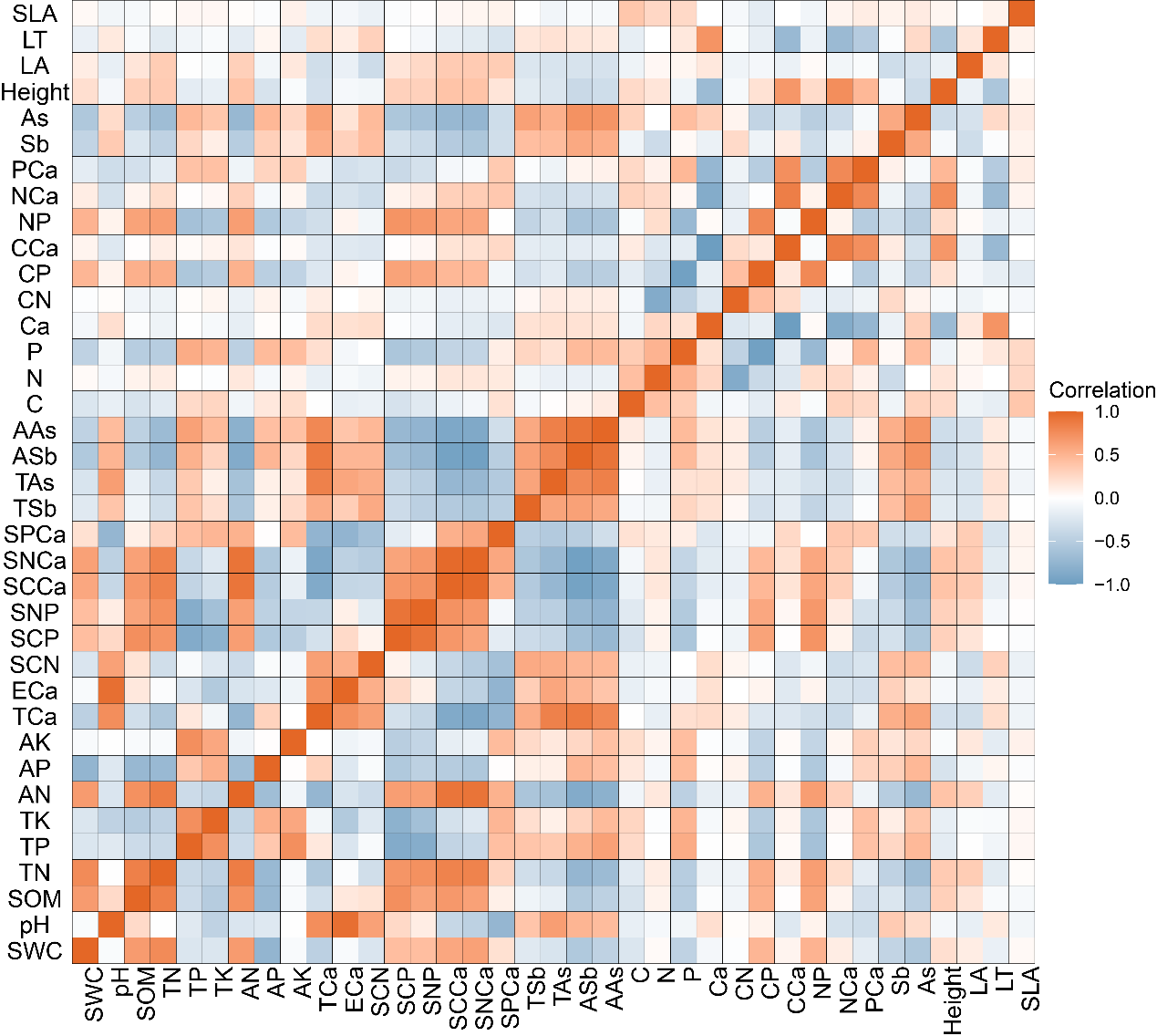
**

**Figure S2** A priori model showing the direct and indirect on the plant metal(loid)s concentration (Pla_Sb/As) driving mechanism from soil physical and chemical properties (Soil_PC), soil metals (Soil_HMs), plant morphological indicators (Pla_MOR), and plant elements (Pla_ELE), and plant multi-element ratio (Pla_RAT).


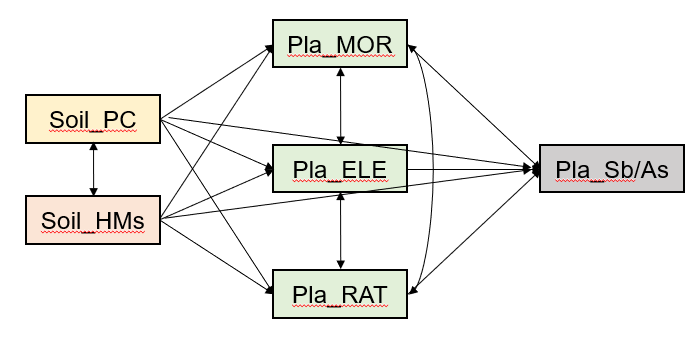


**Figure S3** The Ln*RR* of the aboveground, and underground parts organ carbon (C), nitrogen (N), phosphorus (P), calcium (Ca) concentrations, and its ratio, and antimony (Sb), arsenic (As) concentrations of the four species. The different lowercase of same color indicated significant differences in Ln*RR* among four species (*P* < 0.05). The pot indicates the mean value of Ln*RR*, the line represents the 95% confidence interval (CIs).

**
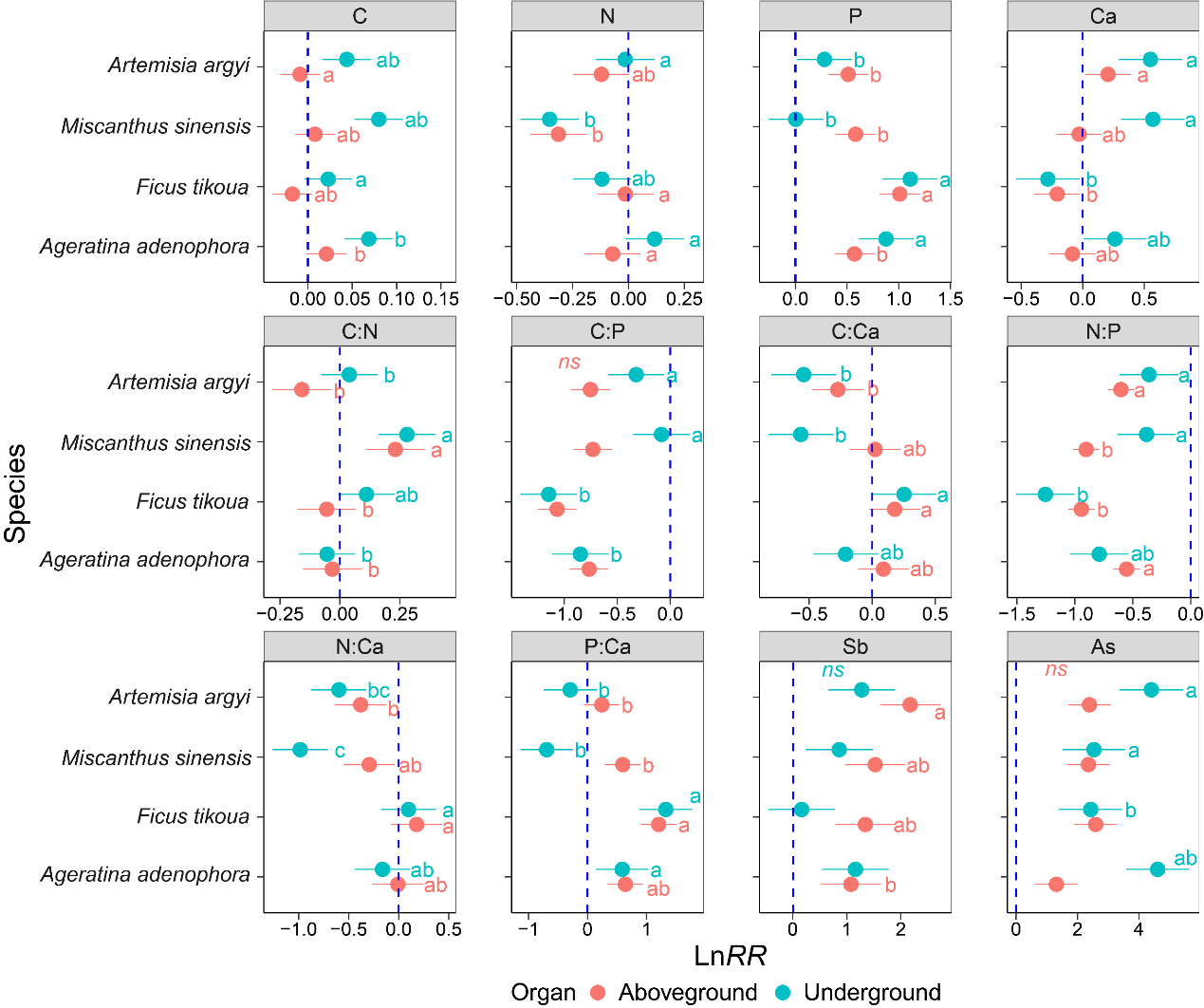
**

**Figure S4** Simple linear regression showed the relationship between C, N, P, Ca and metals (As and Sb) in four species. The adjusted *R*^2^ and *P* values are given in the figure.

**
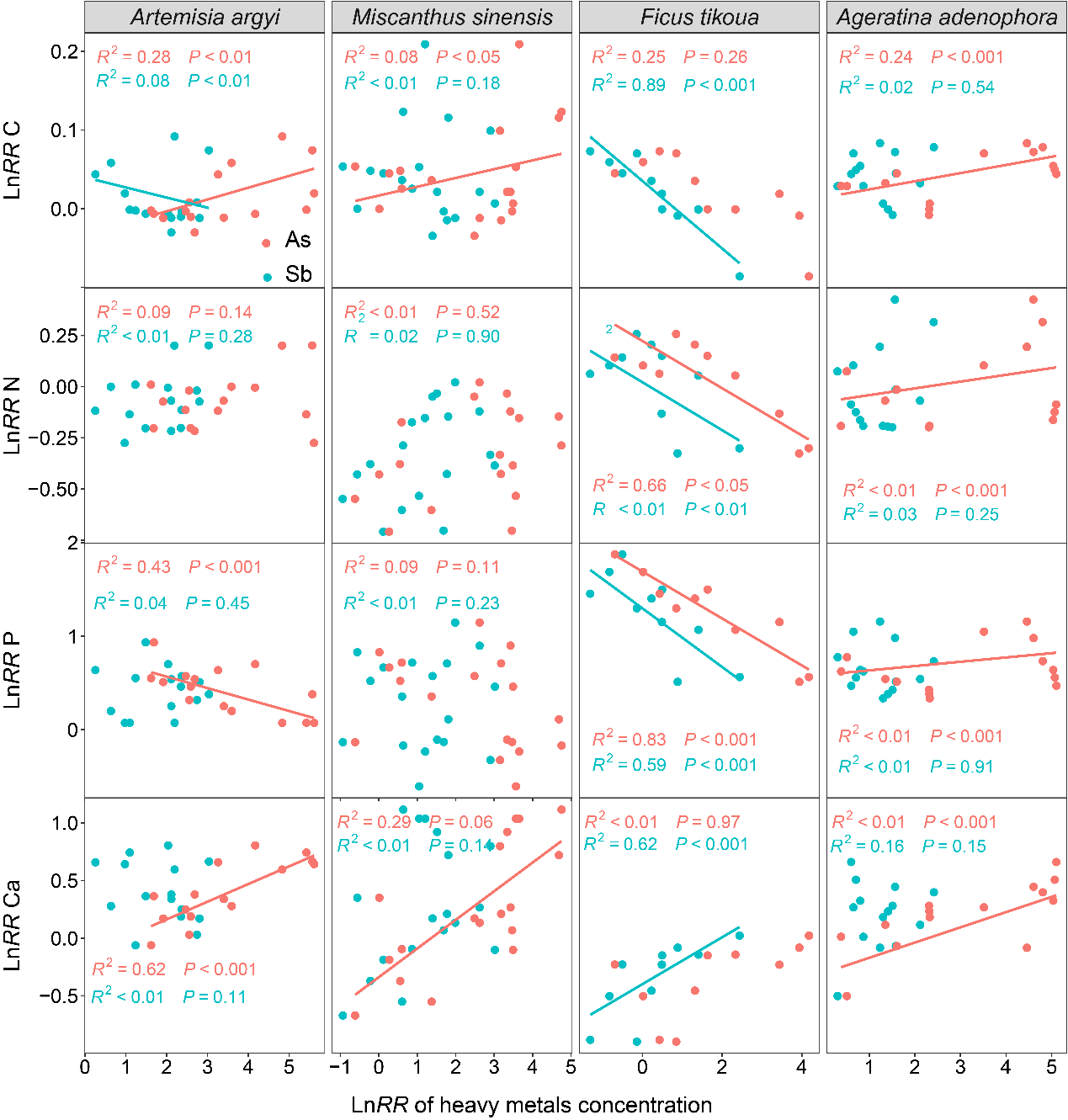
**

**Figure S5** Simple linear regression showed the relationship between ratios of C:N, C:P, C:Ca, N:P, N:Ca, P:Ca and metal(loid)s As, Sb in four species. The adjusted *R*^2^ and *P* values are given in the figure.

**
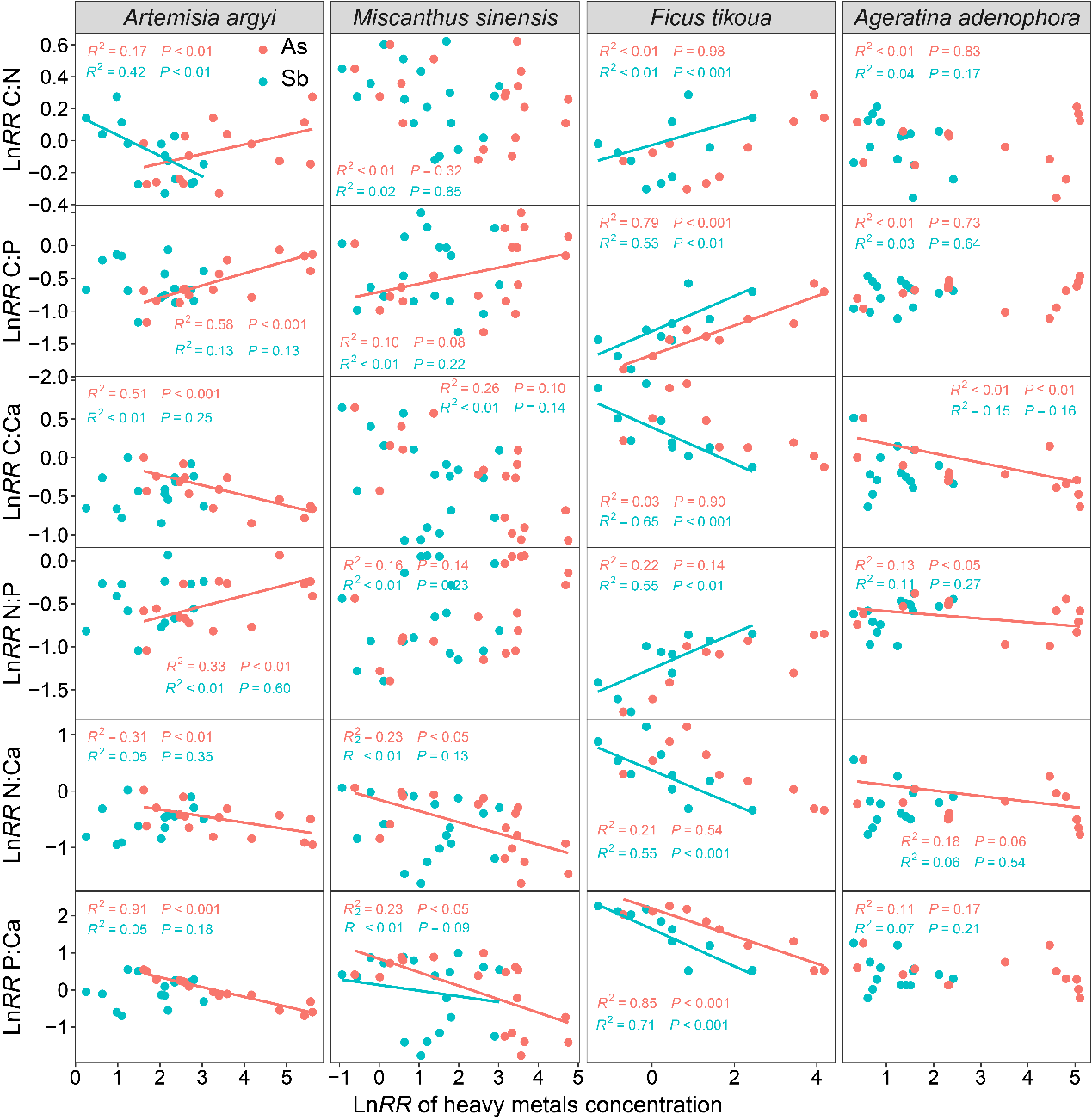
**

**Figure S6** Relationship between Sb and As concentration in all four species. The adjusted *R*^2^ and *P* values are given in the figure. The gray area represents the 95% confidence intervals (CIs).


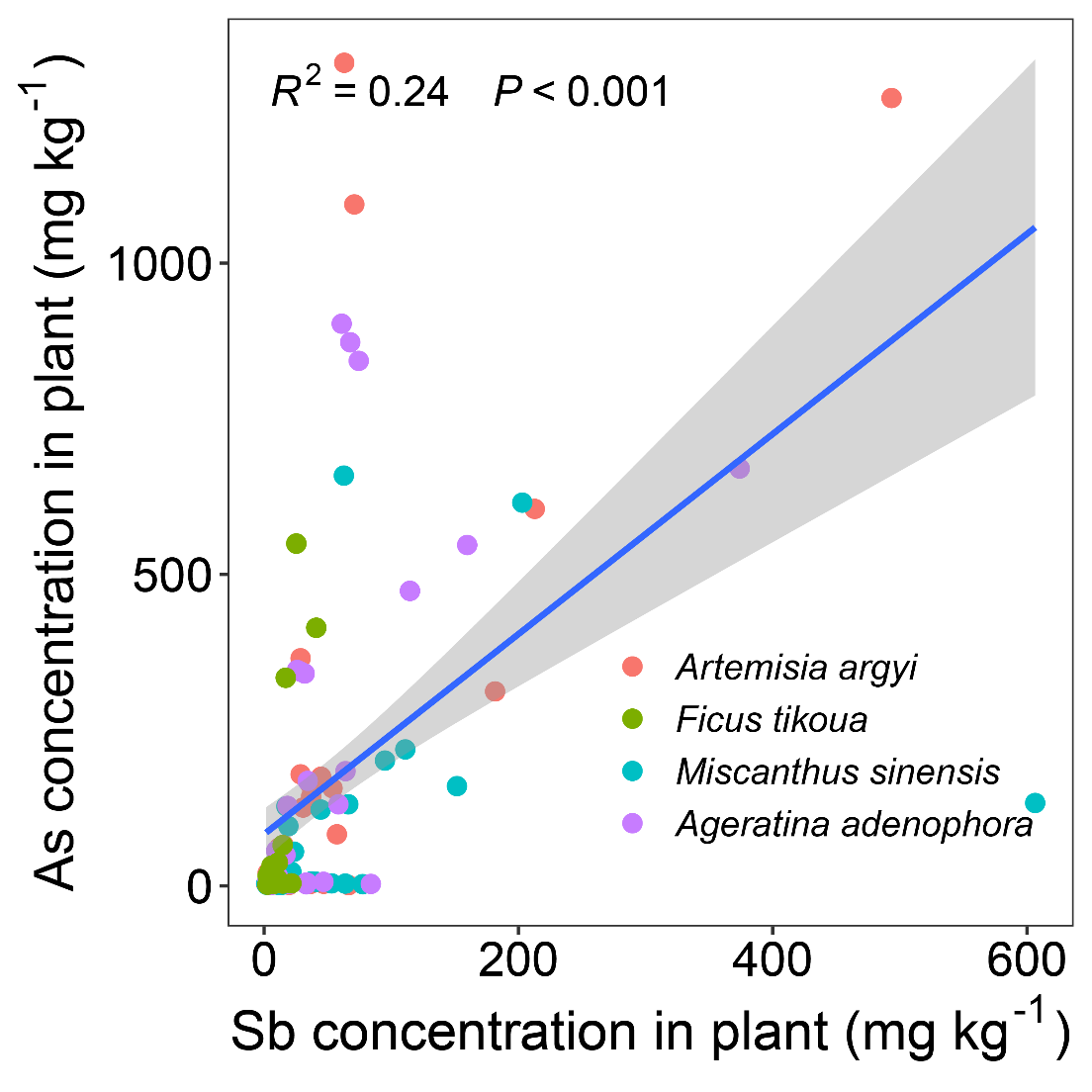


**Figure S7** Relationship between Sb and As concentration in plant of four species. The adjusted *R*^2^ and *P* values are given in the figure. The gray area represents the 95% confidence intervals (CIs).


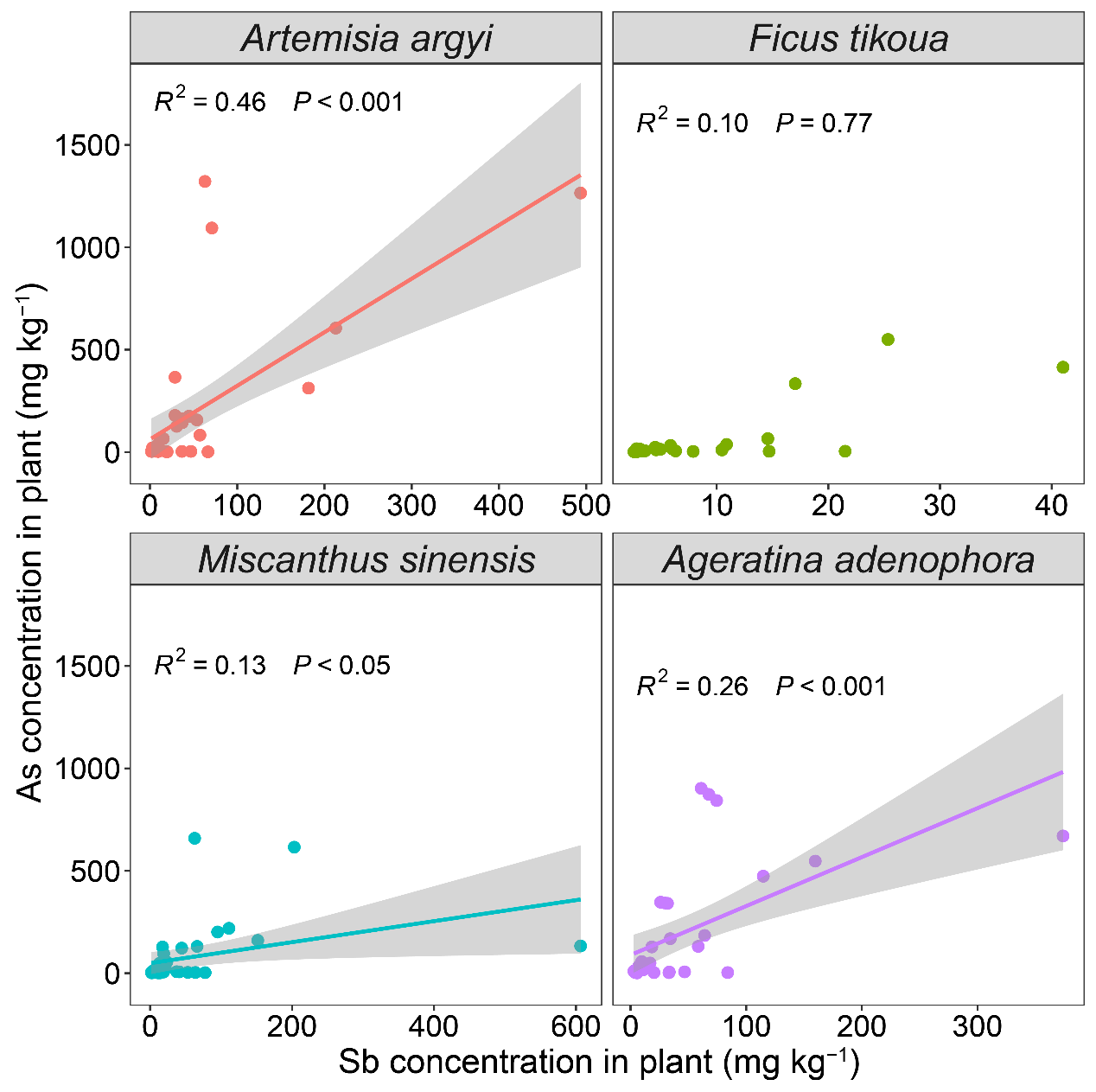


**Figure S8** Principal components analysis (PCA) results of (a) soil physical and chemical properties, (b) soil metal concentrations, (c) plant morphological traits, (d) plant element concentrations and € plant elements concentration ratios.

**
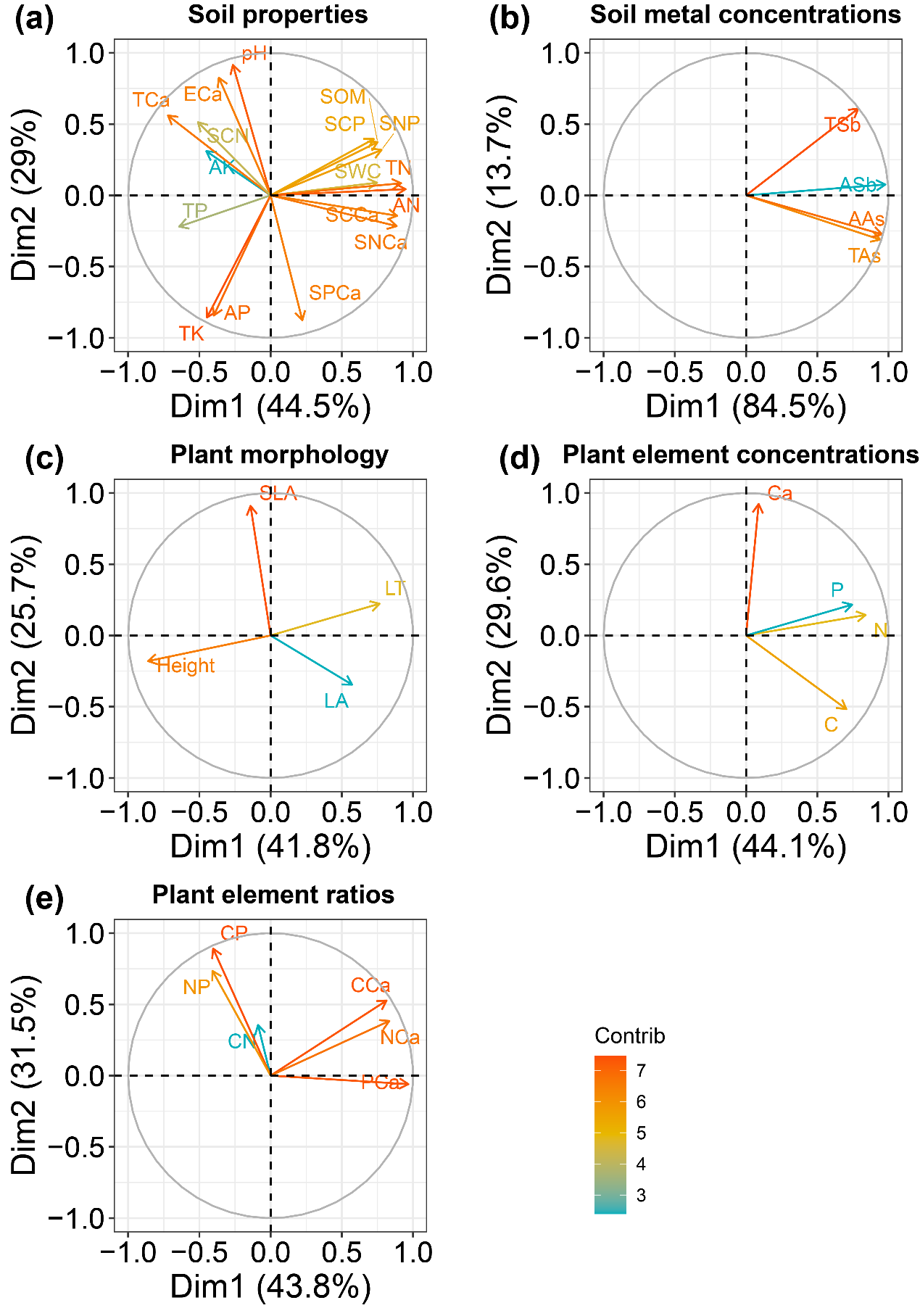
**

**Supplementary Table:**

**Table S1** Principal components analysis (PCA) results of soil physical and chemical properties.

| Factors | PC1 | PC2 |
| --- | --- | --- |
| AK | -0.45** | 0.31** |
| AN | 0.95** |  |
| AP | -0.40** | -0.84** |
| ECa | -0.36** | 0.83** |
| pH | -0.26** | 0.92** |
| SC:Ca | 0.89** |  |
| SC:N | -0.51** | 0.51** |
| SC:P | 0.72** | 0.40** |
| SN:Ca | 0.89** | -0.22** |
| SN:P | 0.78** | 0.32** |
| SOM | 0.75** | 0.37** |
| SP:Ca | 0.22** | -0.88** |
| SWC | 0.75** |  |
| TCa | -0.72** | 0.56** |
| TK | -0.45** | -0.86** |
| TN | 0.92** |  |
| TP | -0.64** | -0.22** |
| Cumulative explanation | 44.52% | 73.55% |

**Notes:** AK, available potassium concentration; AN, available nitrogen concentration; AP, available phosphorus concentration; ECa, exchangeable calcium concentration; SC:Ca, soil organic matter concentration: soil total calcium concentration; SC:N, soil organic matter concentration: soil total nitrogen concentration; SC:P, soil organic matter concentration: soil total phosphorus concentration; SN:Ca, soil total nitrogen concentration: soil total calcium concentration; SN:P, soil total nitrogen concentration: soil total phosphorus concentration; SOM, soil organic matter concentration; SP: Ca, soil total phosphorus concentration: soil total calcium concentration; SWC, soil water content; TCa, soil total caclcium concentration; TK, soil total potassium concentration; TN, soil total nitrogen concentration; TP, soil total phosphorus concentration; **, *P* < 0.01.

**Table S2** Principal components analysis (PCA) results of soil metal(loid)s concentration.

| Factors | PC1 | PC2 |
| --- | --- | --- |
| ASb | 0.98** |  |
| AAs | 0.96** | -0.27** |
| TAs | 0.94** | -0.31** |
| TSb | 0.79** | 0.61** |
| Cumulative explanation | 84.46% | 98.17% |

**Notes:** ASb, soil available antimony concentration; AAs, soil available arsenic concentration; TSb, soil total antimony concentration; TAs, soil total arsenic concentration; **, *P* < 0.01.

**Table S3** Principal components analysis (PCA) results of plant morphological traits.

| Factors | PC1 | PC2 | PC3 |
| --- | --- | --- | --- |
| LT | 0.76** | 0.22** | -0.46 |
| LA | 0.58** | -0.34** | 0.71 |
| Height | -0.86** | -0.18** | 0.01 |
| SLA |  | 0.91** | 0.38 |
| Cumulative explanation | 41.81% | 67.50% | 89.19% |

**Notes:** LT, plant leaf thickness; LA, plant leaf area; Height, plant natural height; SLA, specific leaf area; **, *P* < 0.01.

**Table S4** Principal components analysis (PCA) results of plant elements concentration.

| Factors | PC1 | PC2 |
| --- | --- | --- |
| N | 0.84** |  |
| P | 0.74** | 0.22** |
| C | 0.70** | -0.52** |
| Ca |  | 0.92** |
| Cumulative explanation | 44.06% | 73.70% |

**Notes:** C, plant carbon concentration; N, plant nitrogen concentration; P, plant phosphorus concentration; Ca, plant calcium concentration; **, *P* < 0.01.

**Table S5** Principal components analysis (PCA) results of plant elements ratios.

| Factors | PC1 | PC2 |
| --- | --- | --- |
| P:Ca | 0.97** |  |
| N:Ca | 0.83** | 0.38** |
| C:Ca | 0.81** | 0.53** |
| C:P | -0.40** | 0.89** |
| C:N |  | 0.35** |
| N:P | -0.41** | 0.73** |
| Cumulative explanation | 43.83% | 75.30% |

**Notes:** C:N, plant carbon concentration: plant nitrogen concentration; C:P, plant carbon concentration: plant phosphorus concentration; N:P, plant nitrogen concentration: plant phosphorus concentration; N:Ca, plant nitrogen concentration: plant calcium concentration; P:Ca, plant phosphorus concentration: plant calcium concentration; **, *P* < 0.01.
